# Macroscopic self-reorientation of interacting two-dimensional crystals

**DOI:** 10.1038/ncomms10800

**Published:** 2016-03-10

**Authors:** C. R. Woods, F. Withers, M. J. Zhu, Y. Cao, G. Yu, A. Kozikov, M. Ben Shalom, S. V. Morozov, M. M. van Wijk, A. Fasolino, M. I. Katsnelson, K. Watanabe, T. Taniguchi, A. K. Geim, A. Mishchenko, K. S. Novoselov

**Affiliations:** 1 School of Physics and Astronomy, University of Manchester, Oxford Road, Manchester M13 9PL, UK; 2 Institute of Microelectronics Technology and High Purity Materials RAS, Chernogolovka 142432, Russia; 3 National University of Science and Technology ‘MISiS', Moscow 119049, Russia; 4 Institute for Molecules and Materials,Radboud University, Heyendaalseweg 135, 6525 AJ Nijmegen, The Netherlands; 5 National Institute for Materials Science, 1-1 Namiki, Tsukuba 305-0044, Japan; 6 Centre for Mesoscience and Nanotechnology, University of Manchester, Oxford Road, Manchester M13 9PL, UK; 7 National Graphene Institute, University of Manchester, Oxford Road, Manchester M13 9PL, UK

## Abstract

Microelectromechanical systems, which can be moved or rotated with nanometre precision, already find applications in such fields as radio-frequency electronics, micro-attenuators, sensors and many others. Especially interesting are those which allow fine control over the motion on the atomic scale because of self-alignment mechanisms and forces acting on the atomic level. Such machines can produce well-controlled movements as a reaction to small changes of the external parameters. Here we demonstrate that, for the system of graphene on hexagonal boron nitride, the interplay between the van der Waals and elastic energies results in graphene mechanically self-rotating towards the hexagonal boron nitride crystallographic directions. Such rotation is macroscopic (for graphene flakes of tens of micrometres the tangential movement can be on hundreds of nanometres) and can be used for reproducible manufacturing of aligned van der Waals heterostructures.

## Abstract (web summary)

Precise control of the relative orientation of two two-dimensional layers enables reproducible fabrication of heterostructure devices. Here, the authors show that graphene rotates towards the crystallographic direction of a boron-nitride substrate due to the interplay between van der Waals and elastic energies.

## Graphical abstract

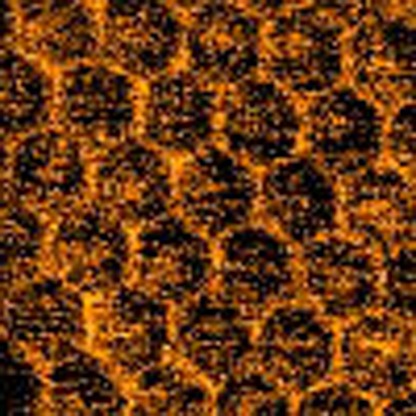

In many layered crystals, it is the van der Waals interaction which is responsible for perfect stacking of individual layers. Once such perfect stacking is lost (for instance through a rotational fault), the van der Waals interaction tends to restore the perfect stacking—the effect known as self-rotation. This effect has been seen for nanometre-sized graphene flakes when driven by an atomic force microscopy (AFM) tip on the surface of graphite^1^. However, up to now, such phenomena has not been observed at micrometre or larger sizes, apart for the cases when such self-rotation was driven by surface free energy in displaced graphite mesa structures^2^,^3^.

One of the reasons why self-rotation is hard to observe in homogeneous systems (where the two surfaces are represented by the same crystals) is because both the self-rotating forces (which try to return the crystals to perfect stacking), and the friction forces are essentially determined by the same van der Waals potential. So, even when close to the perfect commensurate state (where the self-retracting forces should be the strongest), the van der Waals potential would exhibit a number of local potential minima (which correspond to strong friction), where the system may get localized.

The situation is very different when the two crystals are not identical (for instance, have different lattice constants). In this case, the local minima in the van der Waals potential are not expected to play such a significant role, because of strong incommensurability. In addition, if at least one of the crystals has the freedom to relax elastically, the van der Waals potential starts to compete with elastic energy, forming more complex potential landscape. Thus, it is interesting to investigate if the self-rotation can be achieved in such heterogeneous structures.

Such interfaces can be created by stacking several two-dimensional (2D) atomic crystals into van der Waals heterostructures^4^,^5^,^6^, with one of the most interesting systems being graphene on hexagonal boron nitride (hBN)^7^, as the lattice constants of the two crystals are different only by 1.8%. It has been shown that graphene on hBN has an observable moiré pattern, whose period depends on the misorientation angle^8^,^9^. Because of the difference in the interatomic distances for the two crystals, the maximum moiré period (of ∼14 nm) is achieved when the crystallographic lattices are perfectly aligned. At small deviations from the alignment, graphene on hBN undergoes an incommensurate to commensurate transition^10^. In the commensurate state, graphene splits into domains (where its lattice is stretched to gain in van der Waals interaction energy with hBN) separated by sharp domain walls (where graphene lattice is relaxed)^10^,^11^. Within the domain, the stretching is gradual, ranging^11^ from more than 1%, down to 0%. Thus, the average stretching of graphene is quite small (well below 1.8%), resulting in only a small lost in the elastic energy, which is compensated by the gain in the van der Waals energy.

Such stretching of graphene, even so being small, leads to global breaking of the sublattice symmetry^12^,^13^,^14^. Thus, the possibility to align graphene and hBN is extremely important, and already led to the observation of a number of exciting physical phenomena, such as Hofstadter butterfly^15^,^16^,^17^ and topological currents^18^. Furthermore, the concept of self-alignment could be extended to other interfaces and utilized for the formation of novel devices^19^,^20^,^21^,^22^,^23^,^24^,^25^,^26^, which rely on such aligned crystals (for example, resonant tunnelling diodes^27^).

Typically, the commensurate state is identified by a small (of the order of 0.1) ratio between the width of the domain walls (*δ*) and the moiré period (*L*), whereas *δ/L*≈0.5 in the incommensurate phase.

In the following, we demonstrate that, despite the strong competition between the elastic and van der Waals energies, graphene can reorient itself on top of hBN towards a commensurate state (where the crystallographic axis of the two crystals are aligned better than∼0.7°).

## Results

### Demonstration of self-rotation for graphene on hBN

**Figure 1:**
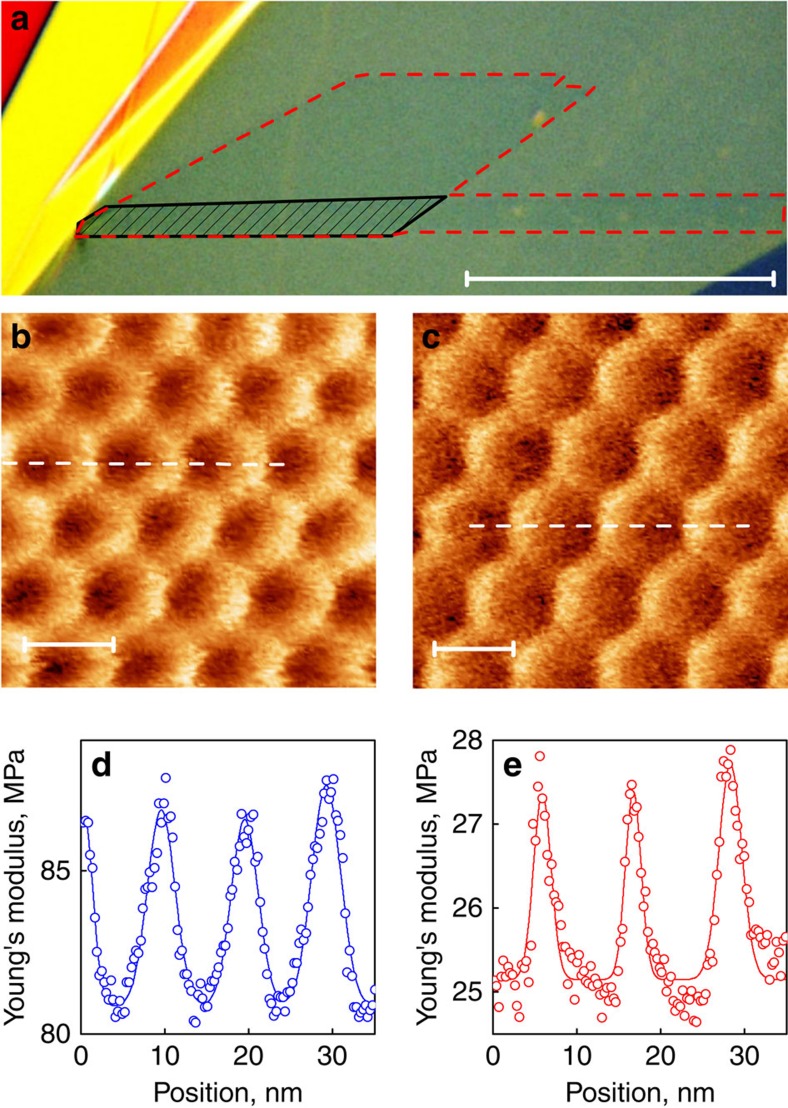
Optical and atomic force microscopy of a self-rotating flake. (**a**) Optical microscopy image of the flake, demonstrating a very clean interface (bubble free) between graphene and hBN (the scale bar is 20 μm). Different colours correspond to different thicknesses of hBN. Graphene is practically invisible and is marked by red dashed line. The hatched area is bilayer graphene. (**b**,**c**) Young modulus distributions obtained in PeakForce Tapping mode of the moiré superlattice before (**b**) and after (**c**) self-alignment. The scale bar in **b** and **c** is 10 nm. (**d**,**e**) Line profiles across the respective Young's modulus distribution images, which indicates the smaller width of the Young's modulus peaks in the annealed (self-rotated) sample. Symbols—experimental data, solid curves—fitted peaks.

**Figure 2:**
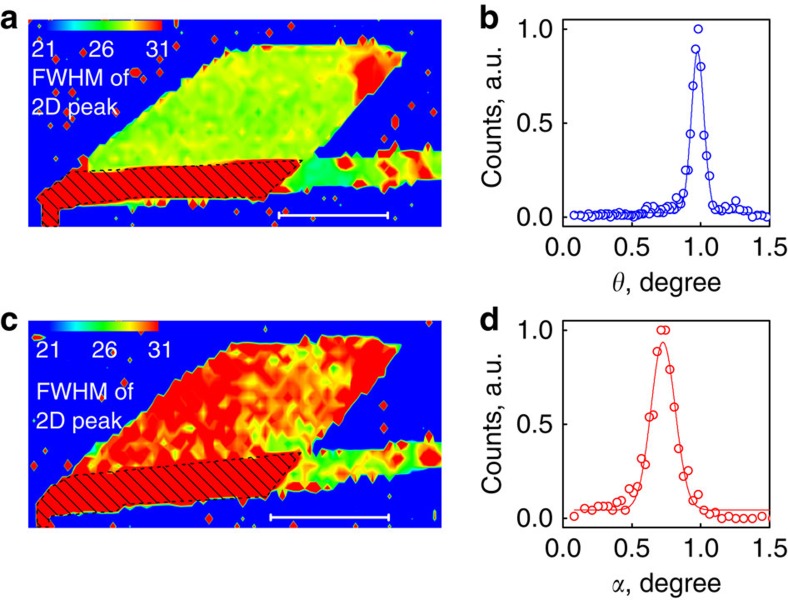
Raman spectroscopy before and after self-rotation. (**a**,**c**) Maps of the full-width at half-maximum of Raman 2D peak before and after annealing, respectively (the scale bars are 10 μm). (**b**,**d**) Histograms of alignment angles, as recalculated from **a** and **c**, respectively.

Graphene flakes, studied in this work, were transferred onto hBN by the dry transfer method^28^,^29^, to produce a clean interface (Fig. 1a). During the transfer procedure, we ensure (by direct optical observation of the crystallographic facets in the transfer set-up) that the crystallographic directions of graphene and hBN are misoriented by *θ*=1–2°. We further confirmed the misorientation angle by measuring the period of the moiré pattern in scanning probe experiments^8^,^9^,^10^ (Fig. 1b) as well as by the width of Raman 2D peak (Fig. 2a), which can be related to the period of the moiré superstructure^30^ and the misorientation angle^30^ (Fig. 2b). Moiré patterns can be observed in various channels in AFM, including topography, friction and so on, as well as in scanning tunnelling experiments^8^,^9^ and conductive AFM^15^. Here we mainly used PeakForce Tapping mode^31^ and evaluated the point Young's modulus channel with a typical resolution better than 2 nm.

Figure 1a shows an optical image of one of our graphene on hBN structures (another example is given in Supplementary Note 1). Originally, it has been aligned by *θ*≈1.0° with respect to the hBN flake, as confirmed by AFM (Fig. 1b) and Raman (Fig. 2a,b). We would like to note that, even before annealing, this flake approaches the commensurate state (*δ*/*L*=0.35, Fig. 1d). The sample was annealed at 200 °C for 4 h in forming gas (90% Ar+10% H_2_). After annealing, *L* increases by 15% (from 10 to 11.5 nm, Fig. 1c), which indicates greater alignment (misalignment angle *θ*≈0.7°). Importantly, *δ*/*L*=0.20 after annealing, which demonstrates an increased level of commensuration (also confirmed by Raman, Fig. 2c,d). The alignment is uniform across the flake, which could be seen from the Raman signal (Fig. 2b,d) or from the observation of the uniform moiré period by AFM measurements in different parts of the sample (see Supplementary Note 2 for details, and similar data for another sample in Supplementary Note 1).

We would like to stress that neither formation of creases nor strain accumulation have been observed after the annealing (as follows from our AFM and Raman measurements, respectively). To achieve such uniform alignment, the graphene flake should have uniformly rotated by Δ*θ*=0.3°. It means that some parts of the flakes should have moved by *d*=Δ*θl*≈0.15 μm (here *l*∼30 μm is the characteristic size of the flake). This is a significant macroscopic movement, which can be used to drive certain nanomachines (such a macroscopic motion is demonstrated in Supplementary Note 1, as well as has recently been seen by other groups as well^32^).

### Theoretical analysis

**Figure 3:**
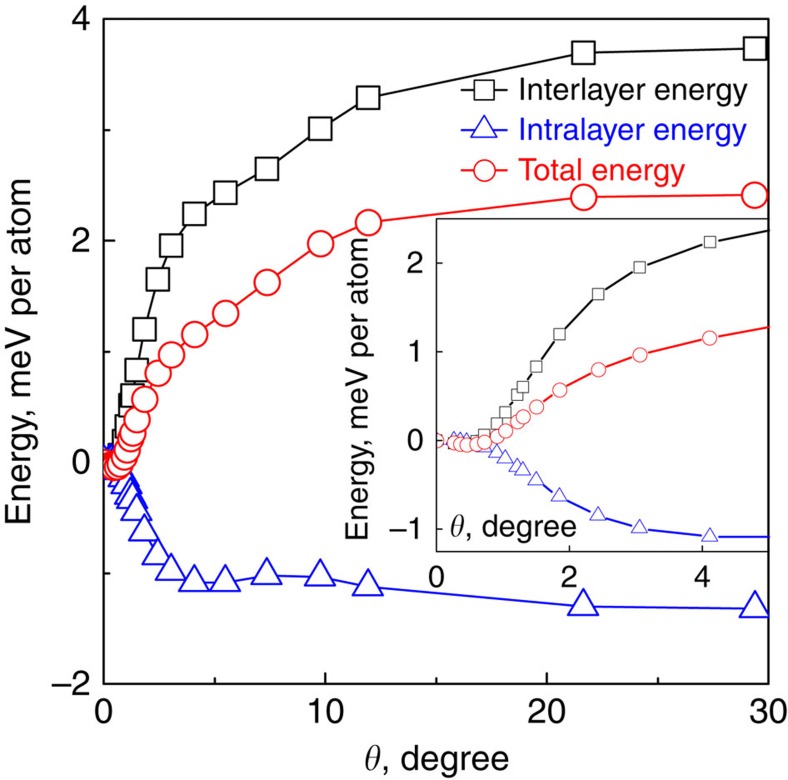
Calculated interaction energies for graphene on hexagonal boron nitride. Total energy (red circles) contributions from intralayer (elastic changes/blue triangles) and interlayer (adhesive/black squares) interactions, as a function of alignment angle, relative to the value at *θ*=0. Points are calculated by minimizing energy for a given angle. Inset: the same curves for low misalignment angles.

What pushes such macroscopic movement is the gradient in the van der Waals forces. To analyse their role, we compare, in Fig. 3, the interlayer van der Waals energy to the elastic intralayer contribution to the total energy after energy minimization for different alignments, relative to the values at 0°. The total energy does not vary up to 0.7°, after which the interlayer energy interaction increases while the intralayer energy decreases, resulting in an increase of total energy. As all values are obtained from energy minimization for a given angle, this figure does not give information about the barriers between different angles.

This picture fits remarkably well with our experimental observation. Our graphene flakes rotated to within 0.7° to the crystallographic orientation of hBN, which corresponds nicely to the plateau in van der Waals energy misalignment dependence for *θ*<0.7°. Still, we note that in many previous experiments^8^,^9^,^10^,^15^,^16^,^17^ the graphene flakes exhibit much better alignment than 0.7°, which we would like to also attribute to the self-rotation mechanism.

### Appearance of one-dimensional wrinkling

**Figure 4:**
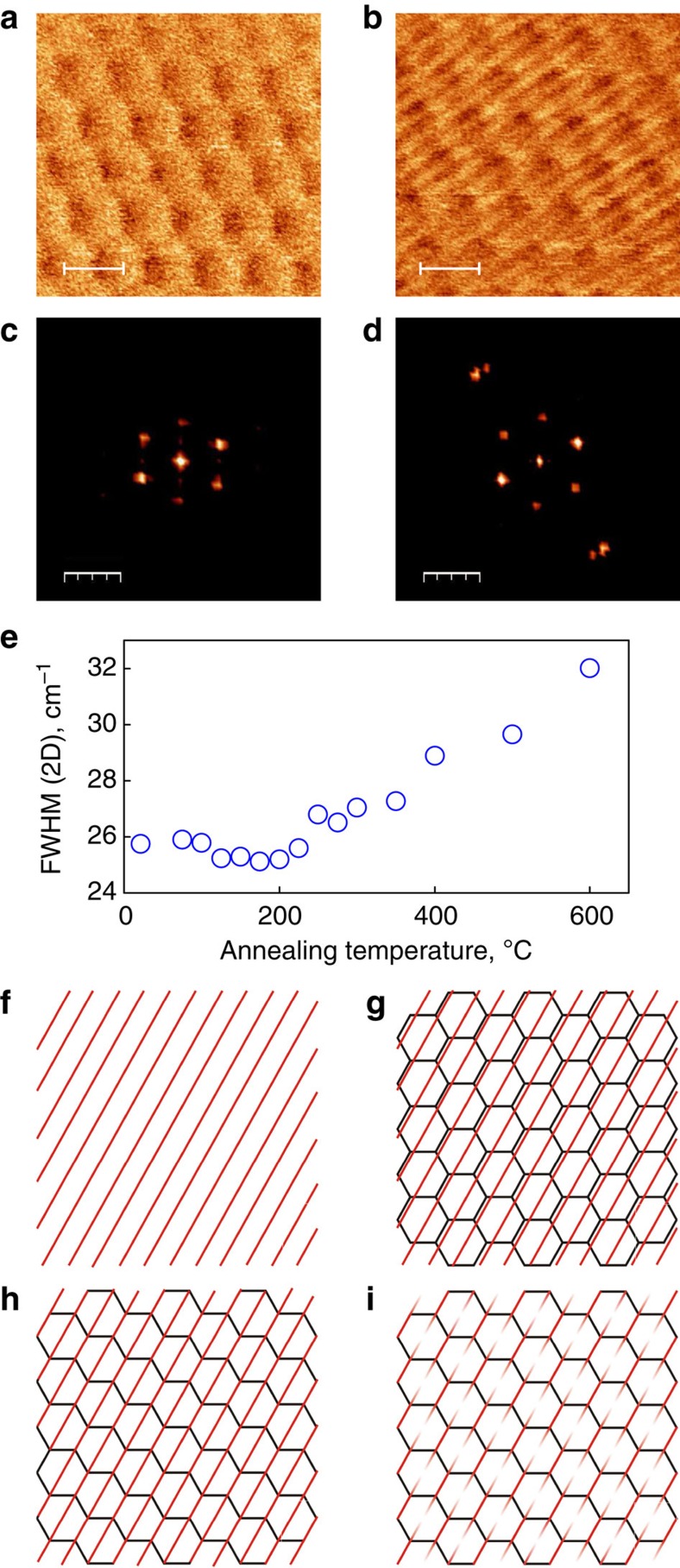
Evidence of uniaxial straining in graphene on hexagonal boron nitride. (**a**,**b**) AFM images of the moiré superstructure in a sample which did not rotate before and after annealing to 600 °C, respectively. The scale bar in **a** and **b** is 10 nm. (**c**,**d**) The Fourier transformations of **a** and **b** are shown, respectively. The scale bar in **c** and **d** is 0.2 nm^−1^. (**e**) Width of the 2D peak in the Raman scattering spectrum as a function of annealing temperature, the increase is linked to the formation of one-dimensional (1D) wrinkles. (**f**–**i**) Proposed structure of the superposition between the moiré pattern and the 1D wrinkles. At high temperatures, 1D wrinkles are formed due to difference in thermal expansion coefficients of graphene and hBN (**f**). Upon cooling, the moiré structure appears, which coexists with the wrinkle (**g**). It is more energetically favourable, however, for the 1D wrinkles to coincide with the domain walls of the moiré structure (**h**). Part of the wrinkle can be flattened because of commensurate–incommensurate transition (**i**).

We would like to stress that not all the flakes become aligned after annealing. We had a number of flakes that do not self-align. At the same time, those which do not undergo the self-rotation would typically form one-dimensional network of wrinkles (Fig. 4b), similar to that reported previously^33^ (although in that case such wrinkles are formed upon cooling). Similar to the moiré pattern, the wrinkles could be observed in several AFM modes, although they are most clearly visible in the local Young modulus and height channels (see Supplementary Note 3 for more examples). The fact that they are readily observable in the Young's modulus channel, suggests strain accumulation around the wrinkles, which is also confirmed by an increase in the full-width at half-maximum of the Raman 2D peak (Fig. 4e; in this case, the broadening is uniaxial, which reflects the fact that wrinkles predominantly create strain only in one direction, see Supplementary Note 4). Figure 4a,b shows the contrasting images in Young's modulus of the moiré pattern before and after annealing to high temperature, respectively. The one-dimensional network of wrinkles is clearly visible on the sample after annealing to be superimposed on the moiré structure (Fig. 4b). At the same time, the period of the moiré structure has not changed. We would also like to suggest that the wrinkles are most likely linked to the moiré structure, as seen from the orientation and the position of the peaks in the Fourier transform patterns.

The proposed mechanism for their formation is the following. At high temperature, because of the difference in the thermal expansion coefficients between the hBN and graphene, the lattice mismatch increases, favouring the incommensurate phase. Upon cooling, the same difference in thermal expansion coefficient acts as a compression for graphene, possibly leading to wrinkles (Fig. 4f). Also, the reconstructed moiré pattern recovers upon cooling, making the two structures (wrinkles and the reconstructed moiré pattern) to coexist (Fig. 4g). However, as both the domain walls of the reconstructed moiré pattern and the wrinkles carry strain field, it becomes energetically favourable to make the two commensurate, overlapping the wrinkles and the domain walls (Fig. 4h). Furthermore, the wrinkles can undergo further reconstruction within the domains of stretched graphene themselves (Fig. 4i). In this model, wrinkles should carry additional strain, which indeed has been observed by strong broadening of the Raman 2D peak (Fig. 4e). Such contribution to the strain energy changes the potential landscape and, as it turns out, prevents the self-alignment process.

Finally, the presence of contamination bubbles and creases seems to prevent the possibility of self-alignment. Self-alignment was not observed in any sample with more than a few bubbles. The detrimental influence of the bubbles can be twofold: it reduces the interaction area between graphene and hBN, making the van der Waals potential landscape shallower; also, the contamination concentrated in such bubbles^29^ can act as pinning centres, preventing any macroscopic movements of graphene.

## Discussion

Our observation opens a new direction in the physics and applications of van der Waals heterostructures—self aligned stacks. Already now this effect is being used to produce graphene aligned on hBN for transport experiments (such as the observation of Hofstadter effect^15^,^16^,^17^, topological currents due to Berry curvature^18^ and so on). In such devices, the self-rotation could be in principle observed directly as shifting of the secondary Dirac point and the associated with it resistance peak (see Supplementary Note 5 for details). We also expect that such self-rotation is not unique to graphene/hBN stacks and that other layered materials should exhibit similar behaviour. For instance, in Supplementary Note 6, we present an example of the observation of self-rotation in graphene/graphene stack being seen via direct measurements of the electronic density of states in tunnelling experiments. Furthermore, one can utilize the mechanical motion of the crystals to produce nanomechanical devices. It is still unclear to what extent the surface reconstruction of the crystal influences the van der Waals potential—a subject still to be explored further both through experimental and theoretical investigations.

## Methods

### Sample fabrication

Our samples were produced by the dry (‘stamp') transfer technique described in detail previously^28^,^29^,^34^. In brief, the method involves using a double polymer layer to identify and isolate graphene flakes on a membrane, before bringing the graphene into contact with the hBN. Importantly, this method does not require the use of any solvents, which minimizes the contamination.

### Sample characterization

AFM measurements were performed on a Bruker FastScan AFM, in the PeakForce^31^ feedback mode, which allows the extraction and analysis of individual force curves for each pixel at regular scanning speeds (0.5–4 Hz). Typically, fast and large area scans are used to determine the period, whereas slower and smaller area scans are used to calculate the ratio *δ*/*L.* Raman spectroscopy measurements were taken with the Witec confocal Raman spectrometer with a wavelength of 514 nm and 1 mW power.

### Details of theoretical analysis

For the calculation of the interaction energies, we constructed a model of graphene on hBN with their crystallographic axis rotated with respect to each other. The hBN is kept fixed to mimic a bulk substrate. Note that a different supercell has to be constructed at each misorientation angle (see Supplementary Note 7 for details). The size of the supercells with periodic boundary conditions demands the use of an empirical potential. The graphene atoms interact through the reactive empirical bond order potential^35^, as implemented in the molecular dynamics code large-scale atomic/molecular massively parallel simulator (LAMMPS)^36^. This potential is widely used in simulations of carbon materials in view of its excellent description of structure and elastic properties of all carbon allotropes. As no potential for graphene/hBN interaction is currently available, the interlayer interaction is assumed to be of the form of a registry-dependent potential for interlayer interactions in graphene^37^, without the correction for bending. We scale this potential to the lattice constant of hBN and use different scaling factors for C–B and C–N interactions as was done in ref. 11 because this leads to a good agreement with experimental results^10^ and *ab-initio* calculations^38^,^39^. We have further refined this approach^13^ leading to a choice of the B–C interaction of 60% of the C–C value, whereas the N–C interaction is set to 200% of the C–C value in the original form^37^. We minimize the total potential energy by relaxing the graphene layer by means of FIRE^40^, a damped dynamics algorithm. For samples close to alignment, this leads to significant changes in bond length along the moiré pattern.

## Additional information

**How to cite this article:** Woods, C. R. *et al*. Macroscopic self-reorientation of interacting two-dimensional crystals. *Nat. Commun.* 7:10800 doi: 10.1038/ncomms10800 (2016).

## Supplementary Material

Supplementary Information Supplementary Figures 1-13, Supplementary Table 1, Supplementary Notes 1-7 and Supplementary References.
