# Supplementary material for: Macroscopic self-reorientation of interacting two-dimensional crystals
Source: Nat Commun. 2016 Mar 10;7:10800. doi: 10.1038/ncomms10800 (PMC4792927; doi:10.1038/ncomms10800)
Supplement: Supplementary Information — Supplementary Figures 1-13, Supplementary Table 1, Supplementary Notes 1-7 and Supplementary References. [file ncomms10800-s1.pdf]

Supplementary Figures

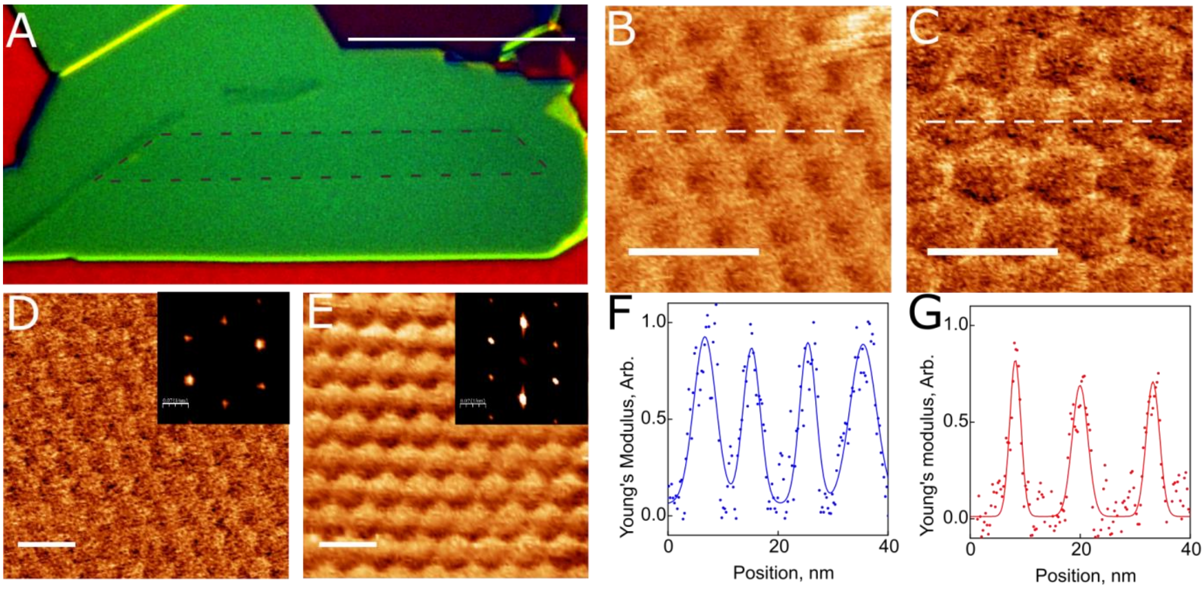

Supplementary Figure 1. Optical and Atomic force microscopy data for a self-rotating flake. *(A) optical microscopy image of the flake showing the clean interface (scale bar is 20 µm), (B) and (C) Young's modulus images of the moiré superlattice before and after self-alignment respectively (Scale from black to white: 60 to 75 MPa, and 45 to 60 MPa), and (D) and (E) larger scale Young's modulus images of the moiré patterns used to extract the periodicity for before and after annealing respectively (Scale from black to white: 60 to 84 MPa, and 40 to 62 MPa). The scale bar in (B), (C), (D), and (E) is 20 nm. (F) and (G) are line profiles of the dashed lines in the Young's modulus maps (B) and (C) respectively. Images (B), (C), (D), and (E) were taken centrally in the right hand region of the flake.*

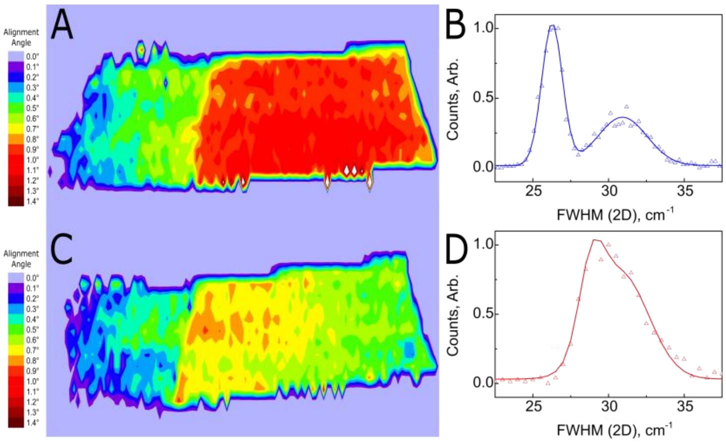

Supplementary Figure 2. Raman spectroscopy data for the self-rotating flake. *(A) and (C) Alignment maps of the flake before and after annealing respectively, taken from the broadening of the 2D peak in the Raman spectrum. (B) and (D) histograms of (A) and (C) respectively.*

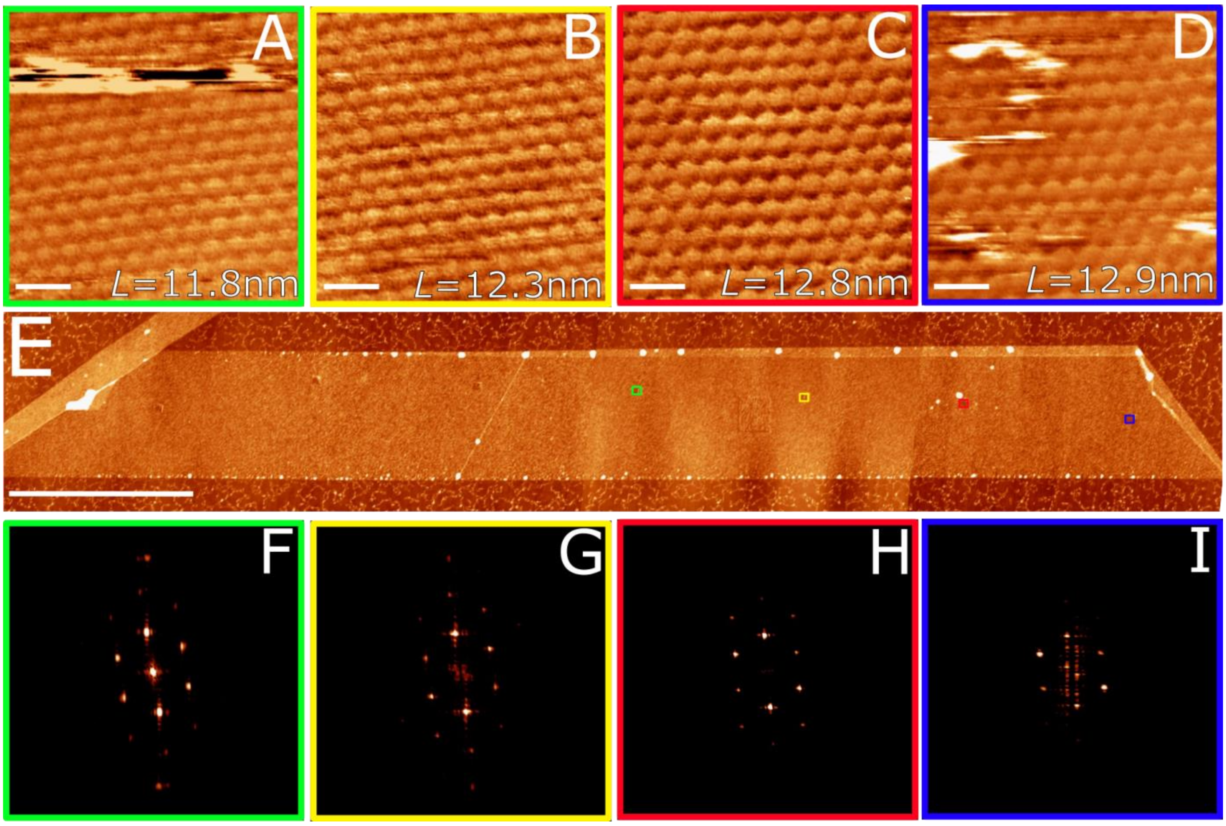

Supplementary Figure 3. A comparison of the moiré pattern at different points on the flake, by AFM. *(A) (Green), (B) (Yellow), (C) (Red), and (D) (Blue) Young's modulus images of the moiré pattern at 5 um intervals along the flakes length (to the right of the fold). These show the gradual change in moiré periodicity. The scale bar in (A), (B), (C), and (D) is 25 nm. Some contamination on the flake surface remains, which is visible in images (A) and (D). (E) an AFM image of the entire flake, with the positions of (A), (B), (C), and (D) indicated by their colours (Also, in order left to right). Also present in the image is the fold which creates a discrete change in relative orientation angles. The scale bar for (E) is 5 µm. (F), (G), (H), and (I) show the Fourier transform of images (A), (B), (C), and (D) respectively. Scales, from black to white, in (A), (B), (C) and (D) are; 119 to 145 MPa, 120 to 140 MPa, 120 to 140 MPa, 110 to 145 MPa.*

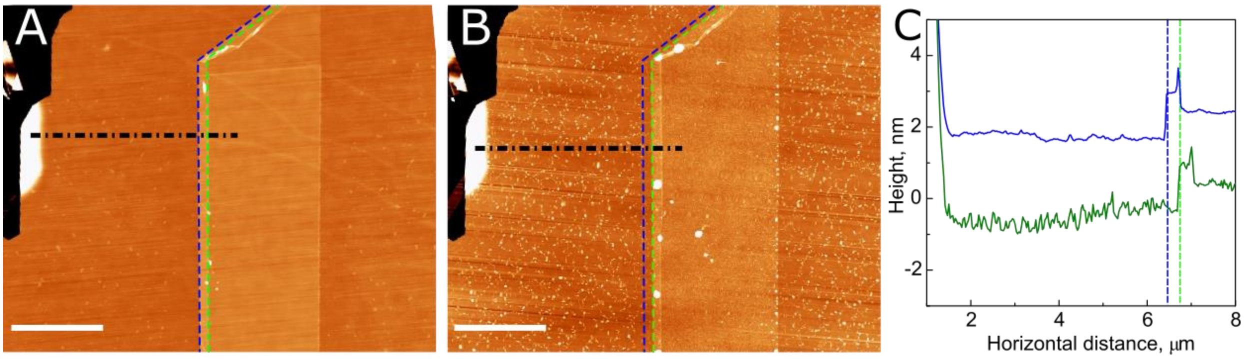

Supplementary Figure 4. AFM data demonstrating the lateral displacement of the flake. *AFM height images of the graphene flake (A) before, and (B) after annealing, with the edge of the flake before (Blue) and after (Green) marked. The scale bar in (A) and (B) is 3 μm. (C) Profiles (averaged over 10 lines) of the black dashed from (A) and (B) line indicating the change in position of the step by 300 ± 90 nm. This step corresponds to the start of the flake.*

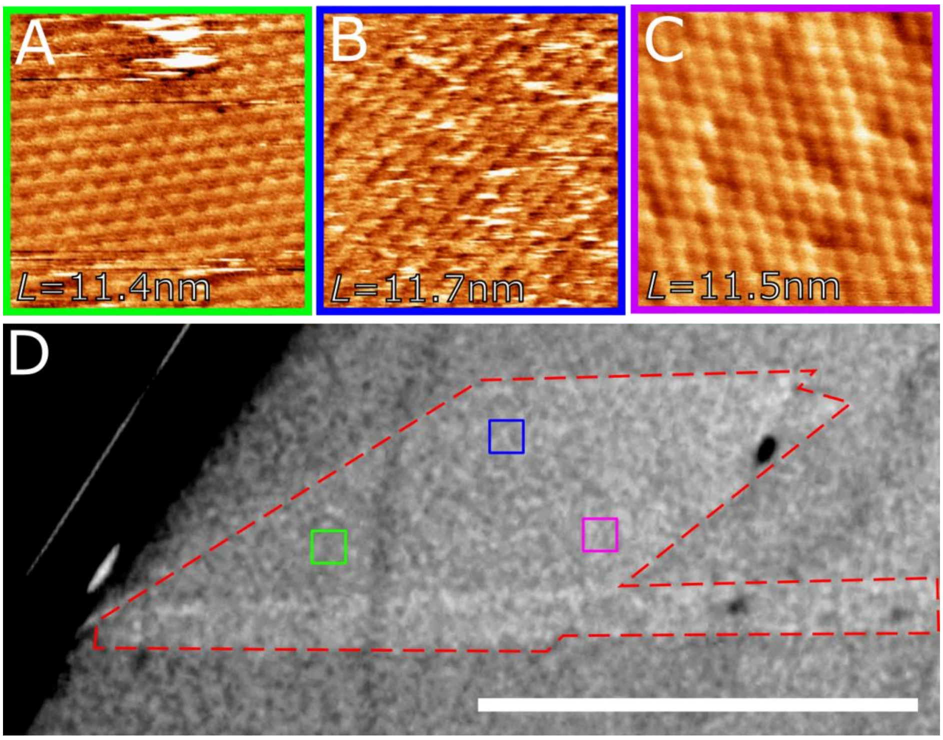

Supplementary Figure 5. AFM data revealing the homogeneity of the moiré pattern. *(A) (Green), (B) (Blue), and (C) (Purple), Young's modulus maps of the moiré pattern at various points on the flake. Each image is 150x150nm. Some contamination remains in the images. Scale from black to white for (A), (B) and (C) is; 31 to 42 MPa, 30 to 45 MPa, and 30 to 45 MPa. (D) Optical image of the flake with approximate regions on the flake for the scans of (A), (B), and (C). The scale bar in (D) is 20 µm.*

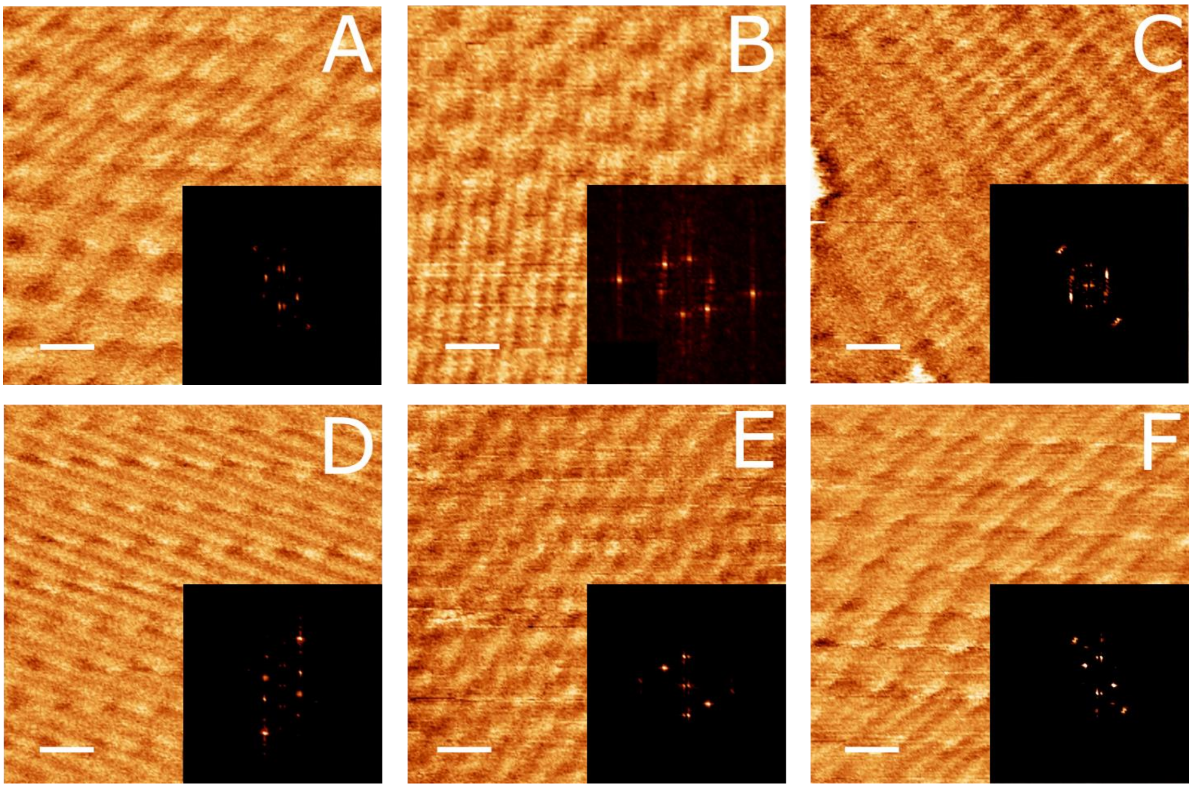

Supplementary Figure 6. 1D wrinkling in various samples. *(A), (B), (C), (D), (E), and (F) Young's modulus maps of the moiré superlattice with the wrinkles also present. In each image the scale bar is 10 nm. Inset to each image is the Fourier transform of the image, which shows the two points associated with the 1D wrinkles. Scales for (A), (B), (C), (D), (E), and (F) are (from black to white); 13 to 19 MPa, 20 to 32 MPa, 100 to 130 MPa, 35 to 49 MPa, 8 to 12 MPa, and 29 to 40 MPa.*

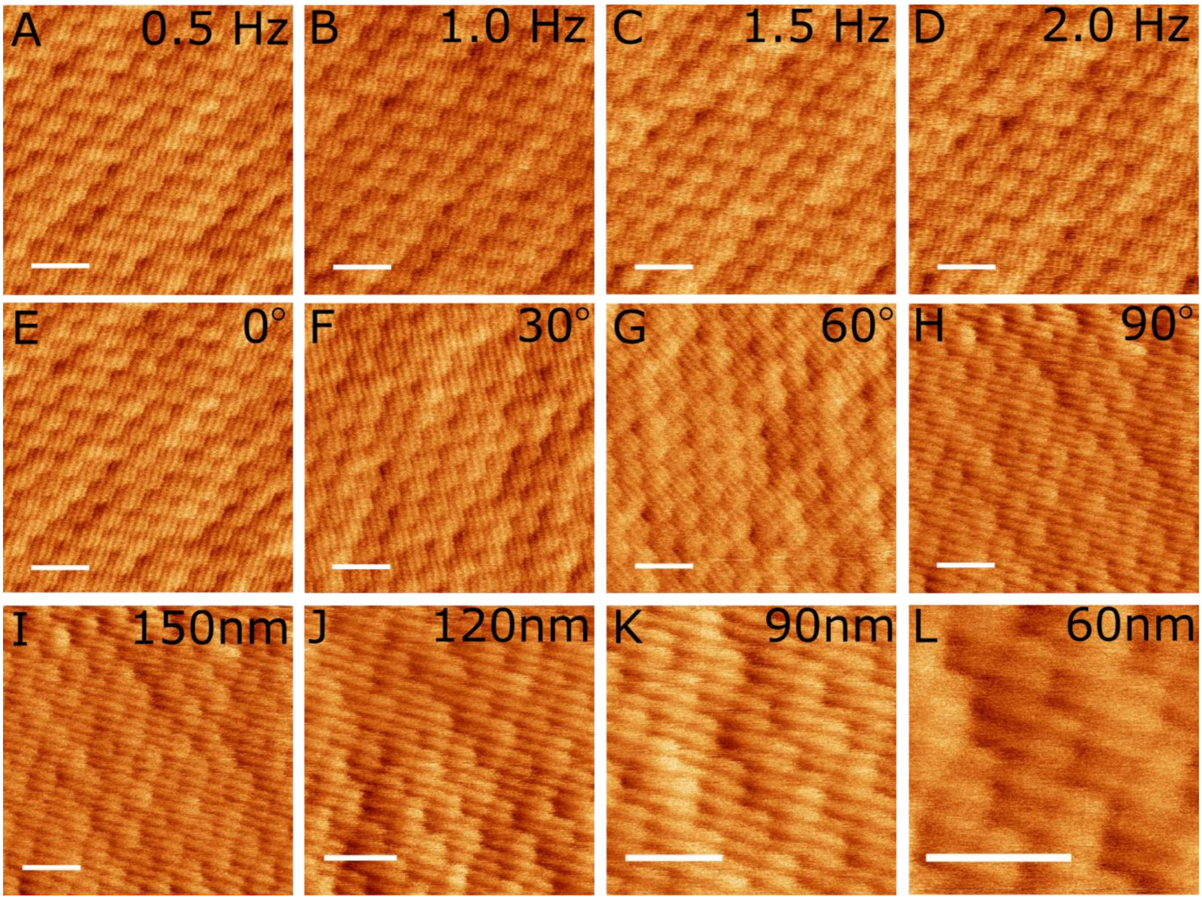

Supplementary Figure 7. AFM data demonstrating that the 1D wrinkling is not an scanning artefact. *(A), (B), (C), and (D) Young's modulus maps showing the periodicity and direction of the 1D wrinkling (and graphene-hBN superlattice) at a scan rate of 0.5 Hz, 1.0 Hz, 1.5 Hz, and 2 Hz respectively. (E), (F), (G), and (H) Young's modulus maps showing that the orientation change of the moiré superlattice and 1D wrinkles for a scan angle of 0°, 30°, 60°, and 90° respectively. (I), (J), (K), and (L) Young's modulus maps showing the moiré superlattice and 1D wrinkles at 150 nm, 120 nm, 90 nm, and 60 nm scan sizes. In each row only one scanning parameter was changed. Some anisotropic lateral drift is present in the images, however it is not significant. The scale bar in each image is 30 nm.*

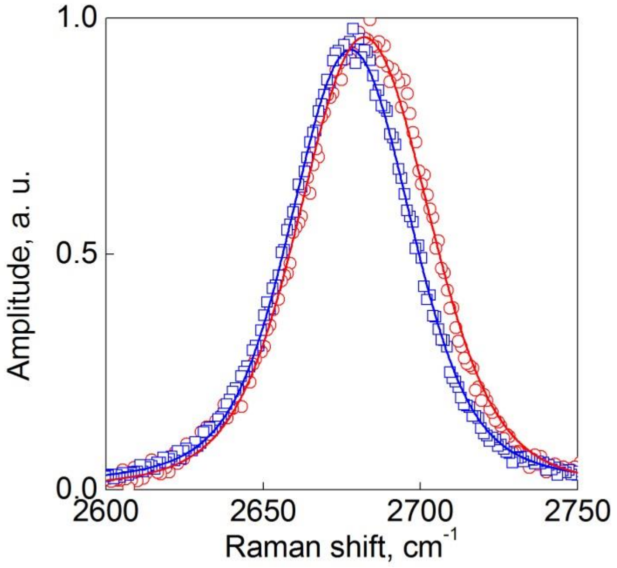

Supplementary Figure 8. Polarised Raman spectroscopy data for 1D wrinkles. *Raman 2D peak for a sample with uniaxial wrinkling (symbols – experimental data, curves - fitting). Red circles and curve – for linear polarization of incoming light perpendicular to the wrinkles, blue squares and curve – for linear polarization of incoming light along the wrinkles.*

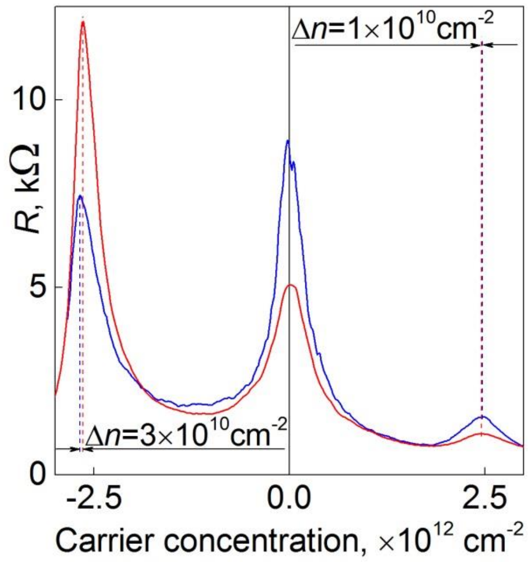

Supplementary Figure 9. Transport data for a self rotating graphene on hBN sample. *Resistance as a function of carrier concentration (measured at 4K) for a graphene on hBN device annealed at 175° (blue curve) and 200° (red curve). The shift in position of the secondary Dirac points indicate self-rotation towards more aligned state.*

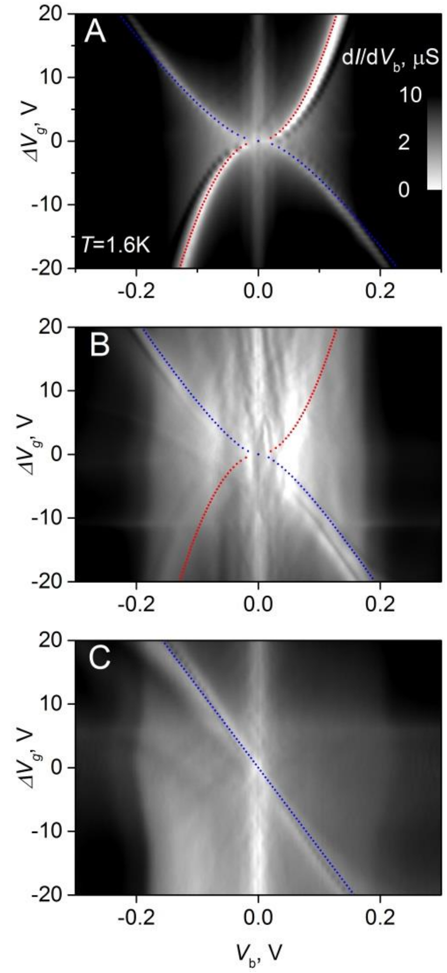

Supplementary Figure 10. Differential conductivity (in logarithmic scale) as a function of the bias and gate voltages for Si/SiO$_2$/graphene/hBN/graphene structure. *(A), Si/SiO$_2$/graphene/hBN/graphene/graphene structure (B) and annealed Si/SiO$_2$/graphene/hBN/graphene/graphene structure (C).The blue (red) curves are the calculated positions of the event when the Fermi level in the bottom (top) graphene electrode passes through the neutrality point.*

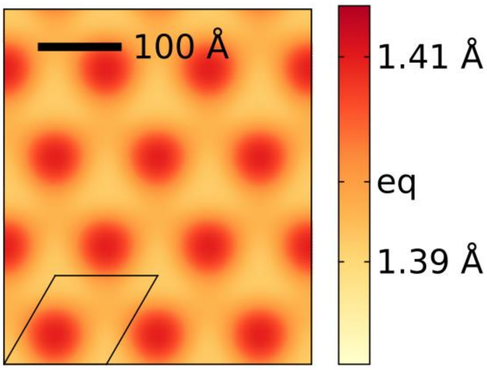

Supplementary Figure 11. Distribution of bond lengths for the relaxed graphene on hBN. *Calculated for θ=0. The equilibrium value given by the REBO potential as implemented in LAMPPS, of isolated graphene, is 1.3978 Å.*

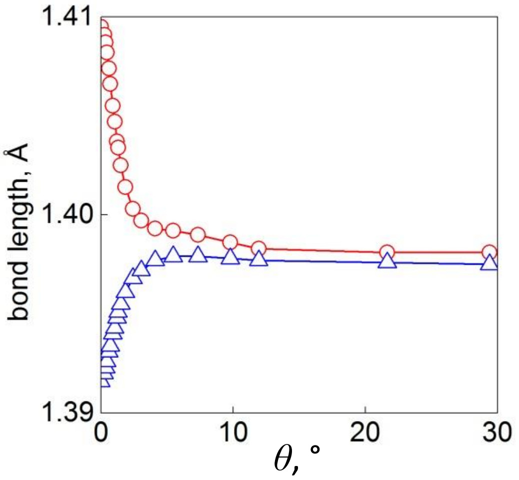

Supplementary Figure 12. Theoretical bond length as a function of misorientation angle. *Maximum (red) and minimum (blue) values of the bond lengths as a function of the misalignment angle* θ.

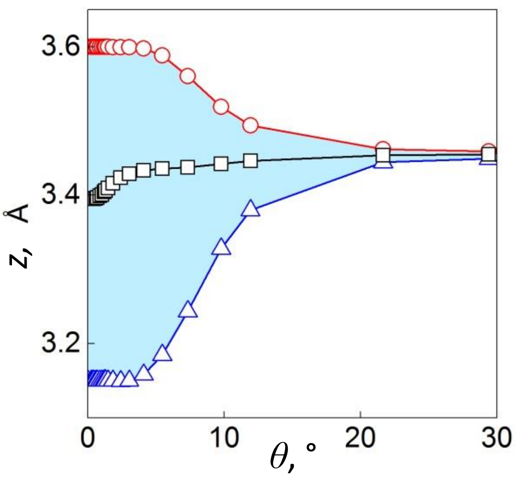

Supplementary Figure 13. Vertical separation between graphene and hBN. *The three lines show the maximum (red), average over all atoms (black), and minimum (blue) values of atomic distances from the substrate as a function of the misalignment angle* θ.

Supplementary Tables

n	m	q	θ
55	0	56	0.0
192	1	196	0.26
137	1	140	0.36
219	2	224	0.45
164	2	168	0.6
272	4	279	0.72
217	4	223	0.91
378	8	389	1.04
161	4	166	1.22
188	5	194	1.3
293	9	303	1.5
105	4	109	1.85
179	9	187	2.43
173	11	182	3.05
185	16	197	4.11
145	17	157	5.48
174	28	193	7.35
122	27	140	9.79
151	42	179	11.94
121	72	172	21.66
56	54	97	29.4

Supplementary Table 1. Parameters used for theoretical calculations. *Parameters n, m and q for our chosen supercells for various misalignment angles* θ.

Supplementary Notes

Supplementary Note 1 – Self rotation

Further to the Flake outlined in the main text, here we demonstrate another flake with self-rotation upon annealing. In this case, a fold in the graphene sheet creates a discrete boundary at which the rotation angle changes w.r.t the hBN substrate even prior to annealing. Supp. Fig. 1A shows an optical image of the flake, supp. The left hand side of the flake is aligned with 0.5° to the hBN substrate as shown by Raman data (supp. Fig. 2A) and AFM data (L=12.5nm), which can be related to each other and the misalignment angle[1-5]. Fig. 3E shows an AFM topography image of the flake, including the fold. The Right hand side of the flake is aligned with an angle of 1° (with L=10nm) to the hBN flake, which is, again, confirmed by Raman spectroscopy (supp. Fig. 2A) and AFM (supp. Fig. 1B, D). It is also partially reconstructed with δ/L=0.4. The Sample has then been annealed to 250°C in a forming gas (Ar 90% and H_2 10%) for 4 hours.

The right hand side of the flake shows considerable change after annealing. Broadening in the Raman spectrum after annealing, displayed in the maps in supp. fig. 2A and C respectively, suggests that the degree of reconstruction within the graphene flake has changed and, interestingly, varies along its length. The moiré periodicity changes, as is shown in supp. Fig. 1D (before) and E (after) to L=12.5nm. This is a change of 25% from L=10 nm before annealing. The flake is also further reconstructed, with δ/L=0.2, whereas prior to annealing in was δ/L=0.4.

As indicated in the Raman map in supp. Fig. 2C, the flake undergoes a novel self-rotation upon annealing. The further from the fold in the graphene sheet, the more the flake has rotated. It varies from L=12.9nm at the very end, to L=11.8nm closer to the fold, which is shown in supp. Fig. 3 (A) – (D). Interestingly, all parts of the flake have changed with respect to the position prior to annealing. The most likely explanation for this variation along the length is that the fold in the graphene sheet acts as a barrier to rotation, whose influence decays with distance.

Finally, further to the high resolution AFM and Raman spectroscopy evidence for rotation, supp. Fig 4 displays the large translation of the graphene flake relative to the hBN substrate. A translation of 300 ± 90 nm is measured from before annealing to after annealing.

Supplementary Note 2 - Flake Homogeneity

Moiré pattern were imaged at various points on the flake (detailed in the main text) to confirm the homogeneity of its period. Supp. Fig. 5A, B, and C show the Young's modulus signal of the moiré pattern at different regions. Unfortunately, some contamination remains on the surface and is visible in (A) and (B). However, there are clean regions, such as (C). The images confirm that the periodicity of the moiré pattern is uniform across the flake.

Supplementary Note 3 - Wrinkled flakes

Many samples show the characteristic wrinkling upon thermal annealing and cooling. Supp. Fig. 6 gives several examples of very pronounced 1D wrinkling on top of the moiré superlattice for several samples which showed no self-rotation. The wrinkles are always linked to the moiré pattern. Similar, corrugation has been seen recently in the graphene-hBN system[6].

Further, to eliminate the possibility that the observed 'wrinkling' is not a result of noise we have performed several basic studies on how it varies with different scanning parameters. Scan angle, scan size, and scan rate have all been changed independently. The results for one sample are displayed in Supp. Fig. 7. Electronic noise and vibrational noise are the two main causes of apparently 1D patterns in raster scanning studies. Since the 1D patterns absolute period and orientation change appropriately with each of these parameters, we can conclude that the observation is of a real physical phenomenon.

Supplementary Note 4 - Uniaxial wrinkling

Our model for unidirectional wrinkling (observed in some of our devices) requires development of uniaxial strain, perpendicular to the wrinkles. In order to test this assumption we performed polarised Raman scattering spectroscopy with the incident light polarisation being parallel and perpendicular to the wrinkles, supp. Fig. 8. We indeed observed that the Raman 2D peak for the polarisation perpendicular to the wrinkles is significantly broadened (FWHM 45.0 cm^{-1}) in comparison to that with polarisation parallel to the wrinkles (FWHM 40.9 cm^{-1}). Note, that no such anisotropic broadening has been observed in the samples which exhibited self-rotation and has not demonstrated wrinkling.

Supplementary Note 5 - Flakes self-rotation: Evidence from transport

Due to electron interference, moiré superstructure results in reconstruction of the electronic spectrum of graphene. In particular, secondary Dirac points appear in the spectrum, which can be detected either through direct measurements of the density of states in STM[1], or in transport, where additional peaks in resistance can be seen when the Fermi level reaches the secondary Dirac points[2,3,7], supp. Fig. 9. The carrier concentration at which the secondary Dirac points is achieved (n_{SDP}) is directly related[3] to the period of the moiré structure as $L=2(\pi/3n_{SDP})^{1/2}$, and could be converted to the misalignment angle[1]. The blue curve in supp. Fig. 9 represents the resistance as a function of the carrier concentration after the initial annealing at 175°C. Converting the position of the resistance peak at the holes side to the misalignment angle we get 0.49°. The red curve was measured on the same sample after an additional annealing at 200 °C. The peaks are shifted towards the lower carrier concentration, which converts to the misalignment angle 0.47°. The small (compared to that presented in the main text) rotation of the flake is associated with the fact that the van-der-Waals potential as a function of the misalignment angle saturates below 0.7° and also with the fact that this graphene flake was clamped by contacts, essential for transport measurements.

Supplementary Note 6 - Self-rotation of graphene on graphene

In order to demonstrate self-rotation of other crystals, beyond the graphene/hBN pair and provide alternative evidences for such rotation, we fabricated Si/SiO_2/graphene/hBN/graphene tunnelling device (tunnelling current is being measured between the two graphene electrodes, 3 layers of hBN serve as a tunnelling barrier and silicon substrate is used as a back gate). The tunnelling differential conductivity as a function of the gate V_g and bias V_b voltages for such a device is presented at Supp. Fig. 10A. The low conductivity features, highlighted by red and blue theoretical curves (see [8] for the details of the electrostatic model which has been used to obtain the two lines), originate from the suppression of the tunnelling when the Fermi level in one of the graphene layers passes through the Dirac point, where the density of states is zero. The specific shape of the features represents the linear spectra of graphene (and the fact that the Fermi energy is a square root function of the carrier concentration).

Then, an additional graphene layer was transferred on the top graphene electrode (so the whole structure now is Si/SiO_2/graphene/hBN/graphene/graphene). We estimate the misalignment

between the two top graphene layers to be approximately 3°. At such misalignment angle the two graphene layers act independently, with the linear dispersion relation still being preserved at low energies in either of them. This fact is confirmed by the observation (Supp. Fig. 10B) of the low-conductivity features (also highlighted by red and blue simulation curves) which are very similar to those observed in Supp. Fig. 10A. Note however, that the green curve in Supp. Fig. 10B is slightly steeper than the blue curve in Supp. Fig. 10A, which reflects the fact that the density of states (still being a linear function of energy) is now doubled for the electrode consisting from two graphene layers.

However, once the sample was annealed at 200°C, the dI/dV_b dependence on V_g and V_b becomes strikingly different, Supp. Fig. 10C. Now only one differential conductivity minimum, which looks like a straight line in the coordinate V_g and V_b is visible (highlighted by blue theoretical curve). We interpret this behaviour as the top graphene layers rotating to form Bernal-stacked bilayer. Since bilayer graphene has a parabolic spectra and, as a consequence, constant density of states as a function of energy, it doesn't contribute with any special features in the conductivity. The minimum in dI/dV_b is originating from the Fermi level in the bottom graphene passing through the Dirac point. The linear behaviour of this line is characteristic of the constant density of states in the top bilayer graphene electrode (here the same electrostatic model as before[8] was used, just the constant density of states for the top electrode was utilised).

Such behaviour demonstrates that similarly to graphene on hBN system, graphene on graphene is also capable to self-rotate into the energetically favourable aligned configuration.

Supplementary Note 7 - Modelling methodology

As the ratio between the lattice constants of graphene and hBN is approximately 55/56, a common supercell can be constructed by repeating 56x56 unit cells of graphene on 55x55 unit cells of h-BN, resulting in a 1.8% mismatch in lattice constant. This construction yields an aligned sample (θ=0) with a 1.8% mismatch of the lattice constants without any initial stretching of graphene.

When lattice relaxation is allowed in the energy minimization - a non-uniform pattern of distortion (Supp. Fig. 11) is found. The spread between local compression and expansion observed in our calculations is from 1.391 Å to 1.41 Å with respect to the equilibrium interatomic distance given by the REBO potential as 1.3978 Å. In supp. Fig. 12, we show the dependence on θ of the maximum and minimum bond-lengths which are almost the same at large angles and show a marked change below 4°. Here we would like to stress that the average (over the unit cell) stretching/compression of

graphene is quite small (well below 1.8%), resulting in only a small lost in the elastic energy, which is compensated by the gain in the van der Waals energy.

To create a common supercell for misaligned graphene on h-BN, we create a supercell which satisfied periodic boundary conditions in the plane for a layer of h-BN rotated by an angle θ with respect to the graphene layer. For some angles, periodic boundary conditions are only satisfied for extremely large supercells. Therefore we allow an adjustment of the lattice constant of graphene of less than 0.01%.

More precisely, we rotate the h-BN by

$$\theta = \frac{1}{2}\arccos\left[\frac{2n^2 + 2nm - m^2}{2\left(n^2 + nm + m^2\right)}\right] \tag{1}$$

resulting in a cell of length

$$a_{cell} = a_{BN}\sqrt{n^2 + nm + m^2} \tag{2}$$

Where a_{BN} is the lattice constant of h-BN and *n,m* are positive integers with *n>m*. The graphene layer is not rotated but the unit cell is repeated *q* times, resulting in a side of length

$$a_{cell} = a_{C'}q \tag{3}$$

where *q* is an integer and the lattice constant of graphene $a_{C'}$ is slightly adjusted to make both supercells the same size. By choosing *n, m* and *q* appropriately, stretching is kept to a minimum (<0.01%).

Details of the studied structures are given in Table I.

The results of energy minimization are shown in the main text. Here we show in supp. Fig. 13, the dependence of the out of plane displacements on the misalignment angle θ. One can see that displacements start occurring below $\theta \approx 15^\circ$ and saturate around 4°.

Supplementary References

1 Yankowitz, M. *et al.* Emergence of superlattice Dirac points in graphene on hexagonal boron nitride. *Nature Physics* **8**, 382-386 (2012).

2 Hunt, B. *et al.* Massive Dirac fermions and Hofstadter butterfly in a van der Waals heterostructure. *Science* **340**, 1427-1430 (2013).

3 Ponomarenko, L. A. *et al.* Cloning of Dirac fermions in graphene superlattices. *Nature* **497**, 594-597 (2013).

4 Woods, C. R. *et al.* Commensurate-incommensurate transition in graphene on hexagonal boron nitride. *Nature Physics* **10**, 451-456 (2014).

5 Eckmann, A. *et al.* Raman fingerprint of aligned graphene/h-BN superlattices. *Nano Letters* **13**, 5242–5246 (2013).

6 Gallagher, P. *et al.* One-dimensional ripple superlattices in graphene and hexagonal boron nitride. arXiv:1504.05253 (2015).

7 Dean, C. R. *et al.* Hofstadter's butterfly and the fractal quantum Hall effect in moire superlattices. *Nature* **497**, 598-602 (2013).

8 Britnell, L. *et al.* Field-effect tunneling transistor based on vertical graphene heterostructures. *Science* **335**, 947-950 (2012).
